# Similar impact of multiple sclerosis and migraine on sexual function in women: Is the multiple sclerosis impact scale questionnaire useful?

**DOI:** 10.1007/s00508-016-1066-x

**Published:** 2016-09-05

**Authors:** Sabine Salhofer-Polanyi, Christian Wöber, Ricarda Prohazka, Assunta Dal-Bianco, Barbara Bajer-Kornek, Karin Zebenholzer

**Affiliations:** 1 Medical University of Vienna, Vienna, Austria; 2 Department of Neurology, Medical University of Vienna, Währinger Gürtel 18–20, 1090 Vienna, Austria

**Keywords:** Sexual dysfunction, Multiple sclerosis, Migraine, Depression, Quality of life

## Abstract

**Background:**

Sexuality is an integral part of overall health but the impact of neurological diseases on sexual function still receives too little attention.

**Aim:**

The aim of this case control study was to compare frequencies and characteristics of sexual dysfunction in women with stable relapsing-remitting multiple sclerosis (MS) and migraine.

**Methods:**

Sexually active women aged 18–50 years were recruited at the MS and headache outpatient clinics of a university hospital and asked to complete questionnaires on sexual function using the multiple sclerosis intimacy and sexuality questionnaire (MSISQ-19) adapted for patients with migraine, depression using the Beck depression inventory (BDI-II) and quality of life using the short form-36 questionnaire (SF-36).

**Results:**

At least one symptom of sexual dysfunction was “almost always” or “always” present in 35.7 % of 42 patients with MS and in 22.6 % of 30 patients with migraine (*p* = 0.3). The MSISQ-19 total score did not differ between the two groups (31.6 ± 10.8 vs. 28.2 ± 11.6, respectively, *p* = 0.2). Sexual dysfunction was categorized as primary, secondary and tertiary in 66.7 %, 40 % and 33.3 % of MS patients and in 57.1 % (*p* = 0.7), 71.4 % (*p* = 0.2) and 71.4 % (*p* = 0.1) of migraine patients, respectively. Depressive symptoms were more common in women with sexual dysfunction than in those without both in MS (*p* = 0.001) and migraine (*p* = 0.006). The SF-36 showed decreasing quality of life with increasing MSISQ-19 sum scores (mental subscale *p* < 0.001 and physical subscale *p* = 0.04).

**Conclusions:**

Sexual dysfunction is a major problem both in women with MS and in women with migraine and is strongly associated with comorbid depression and impaired quality of life. Thus, categorizing sexuality as done by MSISQ-19 is limited by its complex biopsychosocial interactions.

## Introduction

Sexuality as an integral part of overall health is a complex need and easily affected by health-related, psychosocial and emotional conditions. Multiple sclerosis (MS) and migraine are chronic relapsing disorders particularly affecting young and middle-aged women. Although awareness of sexual dysfunction related to MS and migraine has increased in the last few years [1–4] it still remains underestimated and undertreated in daily routine practice.

In MS 30–80 % of the patients experience sexual dysfunctions [1], even in early disease stages when severe disability is absent [5] and quality of life mainly depends on non-motor functions, including a fulfilling sex life [6, 7]. Importance of sexual issues was rated high by MS patients [8], and there is evidence for a detrimental impact of sexual dysfunction on mental aspects of health-related quality of life [6]. Nevertheless, problems are usually not reported on patients own initiative and clinicians interestingly talk about urinary or bladder problems but avoid talking about sexuality [9]. Causes for MS-related sexual dysfunctions are heterogeneous, ranging from causative cerebral or spinal demyelination to disturbing neurological symptoms and psychosocial consequences, negatively interfering with sexual activity [1, 2]. Accordingly, Sanders [10] conceptualized sexual dysfunctions defining them as primary, secondary and tertiary consequences of MS.

For migraine even less data on sexual function are available than for MS. Pathophysiologically, migraine and sexual desire have been linked to 5‑hydroxytryptophan [11]. Clinical studies suggest that the relationship between migraine, sexual desire and sexual activity is much more complex than the cliché of “not tonight honey, I have a headache” [11]. Houle et al. compared 23 migraine patients to 36 with tension-type headache (TTH), found significantly higher sexual desire in migraine sufferers than in patients with TTH and hypothesized that similar serotonergic phenomena may modulate both migraine and sexual desire [11]. In another questionnaire study, one third of 306 migraine patients reported experiences with sexual activity during an attack and 60 % experienced pain relief after sexual activity [12].

A few studies only addressed the question of sexual dysfunction in patients with migraine. Studies in female headache sufferers not including headache-free controls showed sexual dysfunction in 90 % of the patients [4, 13]. This dysfunction was related to depressive symptoms but not to the migraine disability assessment scale score (MIDAS) [14], the frequency and severity of attacks or anxiety [13]. In addition, hypoactive sexual desire disorder was found in 20 % of patients with migraine and/or TTH [4]. Controlled studies confirmed the impact of headache on sexual life: among 44 patients with migraine, 30 with TTH and 30 controls, sexual activity was less frequent and problems in several aspects of sexuality were more common in both headache groups compared to the control group [3]. Among female university students, those with migraine showed a higher health influence on sexual life, higher levels of sexual pain and lower sexual satisfaction compared to women without migraine, whereas sexual activity, desire, orgasm and satisfaction from sexual life did not differ between the two groups [15].

Sufferers from MS and migraine have several features in common. Besides the epidemiological characteristics regarding age and gender, the age at onset and the course of the disease characterized by unpredictable occurrence of bouts or attacks, both disorders have considerable impact on patient life [16]. Physical impairment is not limited to MS but is also a hallmark of migraine considering that even routine physical activity may aggravate a migraine attack. Moreover, physical activity may precipitate an attack or it may be avoided because the patient is afraid of getting or aggravating an attack. Furthermore, both MS and migraine are associated with fatigue [17, 18]. Even though MS is typically considered to be a chronic progressive disorder, this may also be the case in migraine [19, 20]. The most obvious difference between MS and migraine is the degree of physical impairment resulting in the long-term course.

Up to now, there has been no study available comparing the impact of MS and migraine on sexual function in women; therefore, the aim of this study was to evaluate the frequency and characteristics of sexual dysfunction in female patients with stable relapsing-remitting MS and with migraine, to elucidate differences in the impact of these two chronic relapsing neurological diseases and to assess the relation of sexual dysfunction to depression and quality of life.

## Patients and methods

Between 2009 and 2013 women aged 18–50 years were recruited from the outpatient MS and headache clinics of a university hospital and gave written informed consent to participate. Inclusion criteria were a diagnosis of stable relapsing-remitting MS or a diagnosis of migraine according to the criteria of the third edition of the International Classification of Headache Disorders (ICHD-3 beta) [21]. All MS patients were free of migraine and all migraine patients underwent brain magnetic resonance imaging (MRI) during routine diagnostic work-up to exclude cerebral pathologies. Exclusion criteria were other severe physical illnesses, no sexual intercourse during the past 6 months, postmenopause, difficulties to fill in a questionnaire, medication overuse headache, and lack of fluency in the German language. The study was approved by the ethics committee of the Medical University of Vienna.

### Evaluation of sexual function, depression and quality of life (Qol)

Sexual functioning was evaluated by the multiple sclerosis intimacy and sexuality questionnaire (MSISQ-19) [10]. The MSISQ-19 is a self-rating scale, refers to the previous 6 months and includes 19 symptoms of sexual dysfunction rated on a scale ranging from 1 (never) to 5 (always). The maximum sum score is 95 with higher scores indicating more severe sexual dysfunction. An alternative way of analysis is to use a cut-off method: at least one question of the primary, secondary or tertiary domain that was answered with “almost always” or “always” indicates a clinically significant sexual dysfunction.

Looking for a suitable questionnaire on sexual function proved to be the most difficult part. We finally decided to use a questionnaire specific to MS as it was the primary focus of our study and specific questionnaires on sexual function in migraine do not exist. In our opinion, non-disease specific questionnaires, such as the female sexual function index [22] or the Arizona sexual experiences scale [23] pay too little (or no) attention to the importance of symptoms associated with a chronic disease; however, there are several similarities between MS and migraine, which supported our decision but made it necessary to adapt the MSISQ-19 for suitability in patients with migraine. In detail, we asked migraine patients to think about migraine attacks or precipitating symptoms when answering the following questions: feeling of dependency due to migraine, feeling less feminine due to migraine, exacerbation or significant worsening of migraine symptoms, feeling less confident about sexuality due to migraine and fear of being rejected due to migraine. The concept of sexual dysfunction defined as primary, secondary or tertiary remained unchanged for both MS and migraine patients. Primary sexual dysfunctions referred to impairment of sexual response or sexual feelings (e.g. altered genital sensation, decreased libido, problems with arousal and orgasm and reduced vaginal lubrication) in MS caused by demyelination and in migraine by altered serotonergic function. Secondary sexual dysfunctions refer to changes that indirectly impair sexual response, such as pain, cognitive or concentration problems. Tertiary sexual dysfunctions are caused by disability-related psychological, emotional, social and cultural influences that may interfere with sexual functioning (e.g. altered self-image, lowered self-esteem, anger, feeling of rejection, feeling less feminine, isolation, guilt, change in gender roles and feelings of dependency). Of the 19 items, 5 items refer to primary, 9 to secondary and 5 items to tertiary sexual dysfunctions.

Quality of life was evaluated by short form 36-questionnaire (SF-36) [24] and depression by the Beck depression inventory II scale (BDI-II) [25]. The SF-36 is a self-rating questionnaire for evaluating health-related quality of life (QoL). Subjects have to rate their QoL during the preceding 4 weeks. Raw scores are transformed into scores between zero (lowest QoL) and 100 (best possible QoL). The questions can be grouped into eight categories (subscales) and in addition summed scores for a physical and mental component can be calculated [24]. The BDI-II is also a self-rating questionnaire with 21 items and a maximum summed score of 63 with higher summed scores indicating more severe depression [25].

## Statistical analysis

Descriptive statistics (mean ± standard deviation, median or frequencies and percentages) are given for all variables. For comparison between groups, parametric tests were used (t-test). Fisher’s exact test was used for proportions and Spearman’s rank test for correlation analysis. Analysis was performed using SPSS 20. All *p*-values <0.05 were considered statistically significant.

## Results

### Demography

We screened 82 female patients of which 6 patients had no sexual intercourse within the previous 6 months, 3 additional patients were excluded due to incomplete questionnaires and 1 because of pure TTH. A final number of 42 MS patients with a median EDSS of 1.75 and 30 patients with migraine were included for further analysis. Of the migraine patients five had an additional diagnosis of TTH. Table 1 provides more demographic characteristics.

**Table 1:** Demographic characteristics of patients with multiple sclerosis and migraine

Parameter	Multiple sclerosis *n* = 42	Migraine *n* = 30
Mean age, years (SD)	34 (7)	32 (9)
Median EDSS	1.75	–
*Family status*		
Single	40.5 %	27.6 %
Married/cohabitation	45.2 %	62.1 %
Divorced	14.3 %	6.9 %
Widowed	0 %	3.4 %
*Children*		
Yes	35.7 %	48.3 %
No	64.3 %	51.7 %
*Occupation*		
Apprentice/student	14.3 %	0 %
Employed	52.4 %	27.5 %
Self-employed	2.4 %	0 %
Housewife	4.8 %	72.4 %
Retired	16.7 %	0 %
Unemployed	9.5 %	0 %
*Education*		
Compulsory education	2.4 %	3.4 %
Apprenticeship/secondary school graduation	69 %	79.3 %
University	28.6 %	17.2 %

*SD* standard deviation, *EDSS* Expanded disability status scale

### Sexual dysfunction

Using the cut-off method 35.7 % of MS patients and 22.6 % of headache patients had some form of sexual dysfunction, i. e. at least 1 out of the 19 symptoms of sexual dysfunction was present “almost always” or “always” (Fisher’s exact test *p* = 0.304). Sexual dysfunctions were categorized as primary, secondary or tertiary in 66.7, 40, and 33.3 % of MS patients and in 57.1, 71.4 and 71.4 % of headache patients, respectively. Thus, numerically, the prevalence of primary dysfunction was similar in MS and migraine, whereas secondary and tertiary dysfunctions were more common in migraine. None of the differences, however, were statistically significant (Fisher’s exact test: *p* = 0.7, *p* = 0.2, *p* = 0.1, respectively). Combinations of primary, secondary and tertiary sexual dysfunctions were also numerically more common in patients with migraine but again the differences were statistically not significant (33.4 % vs. 71.5 %, Fisher’s exact test *p* = 0.2).

The mean MSISQ-19 summed score was 31.6 ± 10.8 (range 19–69) in MS and 28.2 ± 11.6 (range 19–69) in migraine (t-test *p* = 0.2). Table 2 contains further details on sexual dysfunctions in patients with MS and migraine.

**Table 2:** Characteristics of sexual dysfunction in patients with multiple sclerosis and migraine

	Multiple sclerosis *n* = 42	Migraine *n* = 30	*p*-value
Any form of sexual dysfunction (SD)	35.7	22.6	0.304^a^
Combined SD	33.4	71.5	0.172^a^
MSISQ-19 summed score (SD)	31.6 (±10.8)	28.2 (±11.6)	0.204^b^
*Primary SD* Altered genital sensation Reduced libido Problems with arousal Problems with orgasm Reduced lubrication	66.7 13.3 46.7 26.7 40 27.7	57.1 28.6 42.9 28.6 14.3 28.6	0.665^a^
*Secondary SD* Muscle tension or spasticity Bladder problems Bowel problems Shaking of hands or body Pain or burning sensations in the body Problems with body control Memory or concentration problems Feeling of dependency Relapse/migraine attack	40 6.7 13.3 0 0 0 6.7 28.6 13.3 20	71.4 14.3 0 14.3 0 14.3 0 42.9 28.6 14.3	0.170^a^
*Tertiary SD* Feeling unattractive Feeling not feminine Fear of rejection by partner Fear of dissatisfying partner Lowered self-esteem	33.3 20 6.7 6.7 6.7 20	71.4 71.4 0 0 28.6 0	0.095^a^

For comparison between groups two-sided t‑test was used and Fisher’s exact test was used for proportions

^a^Fisher’s exact test

^b^Two-sided t‑test

### Severity of the disease

The EDSS score showed a statistically significant positive correlation to the MSISQ-19 summed score (Spearman’s rank correlation *p* = 0.02).

### Depression and quality of life

The prevalence and severity of depressive symptoms assessed by BDI-II was similar in patients with MS and migraine (Fisher’s exact test *p* = 0.847). Symptoms were absent in 47.6 % and 48.4 %, minimal or mild in 35.7 % and 32.2 %, moderate in 9.5 % and 6.5 % and severe in 7.1 % and 9.7 %, respectively (Fisher’s exact test *p* > 0.05).

Analyzing the entire group of 72 patients, depression was significantly more common in patients with sexual dysfunction (90.9 %) than in those without (34 %, Fisher’s exact test *p* < 0.001) and there was a positive correlation of BDI-II and MSISQ-19 summed scores (Spearman’s rank correlation *p* < 0.001). Depression was also significantly associated with sexual dysfunction when using the cut-off method and analyzing data separately for MS (Fisher’s exact test *p* = 0.001) and migraine (Fisher’s exact test *p* = 0.006). Prevalence rates of depression in women with and without sexual dysfunction were 93.3 % and 29.6 % in MS (Fisher’s exact test *p* = 0.001) and 85.7 % and 39.1 % in migraine, respectively (Fisher’s exact test *p* = 0.006).

With respect to quality of life, the physical summed score of SF-36 and the pain score were higher in MS than in migraine (t-test: physical sum score 48 vs. 42, *p* = 0.035, pain score: 74 vs. 39, *p* < 0.001, respectively) indicating that patients with MS experienced better physical quality of life and less pain than patients with migraine. In contrast, the mental summed score, as well as the other subscales of SF-36, did not show statistically significant differences between MS and migraine. In both groups, the physical and mental summed scores showed a statistically significant negative correlation with the MSISQ-19 summed score (Spearman’s rank correlation MS *p* < 0.001, migraine *p* = 0.04).

## Discussion

This is the first cross-sectional case control study comparing the impact of stable relapsing-remitting MS and migraine on sexual function in women. Our study suggests that sexual dysfunction is a major problem both in women with MS and with migraine. More than one third of the patients with MS and almost one quarter of those with migraine had clinically relevant sexual dysfunction, i. e. they experienced at least 1 out of 19 symptoms of the MSISQ-19 almost always or always during the preceding 6 months. The difference was statistically not significant. Similarly, the two patient groups did not differ with respect to primary, secondary or tertiary sexual dysfunction. In the entire group of patients and in the two subgroups, sexual dysfunction was related to depression and impaired quality of life.

Both MS and migraine show female preponderance and patients share the burden of suffering from a chronic recurrent neurological disease with unpredictable clinical exacerbations (i. e. relapse vs. migraine attack) accompanied by and leading to further disease-related problems between attacks, only one of which being sexual dysfunction. Even though sexual dysfunctions, such as hypoactive sexual desire and sexual pain were described in headache patients [4, 15], our finding that MS and migraine patients were equally affected at all levels was remarkable.

Considering the differences in the nature of MS and migraine, we expected to find significantly more primary and secondary sexual dysfunctions in MS and tertiary dysfunctions in both MS and migraine patients. Of the headache patients four (all with normal brain MRI) actually reported sexual dysfunctions, such as reduced lubrication, problems with arousal and orgasm or reduced libido, all of them classified as primary according to Sanders’ concept [10], thus pointing to its limitations. Categorizing sexuality into levels is limited by its complex biopsychosocial interactions. Even in MS, sexual dysfunctions defined as primary may not always be the result of underlying pathophysiological central nervous system (CNS) changes but more a direct consequence of unhappy partner relationships, other psychological or social influences or depression [26–28]. Anhedonia, a depression-specific symptom, is related to lowered sexual desire, thus probably mimicking primary sexual dysfunction. Indeed, a strong association of depression and sexual dysfunction was apparent in our study with only two patients suffering exclusively from sexual dysfunction without concomitant depression. Similar results underlining the major negative impact of depression on sexuality have been previously published [4, 13, 29–31]. A study focusing on sexual dysfunctions in chronic diseases found that comorbid depression was frequent and independently determined the presence of sexual dysfunction [32]. Interestingly, former studies using MSISQ-19 either did not assess depression by a specific questionnaire [6, 33, 34] or correlation analysis was only done between depression and tertiary sexual dysfunction [5]. Furthermore, to the best of our knowledge there are no MRI studies supporting Sanders’ concept of primary sexual dysfunctions due to causative cerebral demyelination. Considering all these points, the equal prevalence of the three domains of the MSISQ-19 in MS and migraine give rise to doubts about the usefulness of the concept behind the MSISQ-19. Additionally, the prevalence of sexual dysfunction in MS is associated with higher EDSS scores [29, 31]. In this study, patients were not severely disabled (median EDSS 1.75), even had a better perception of their physical health and experienced less bodily pain than headache patients, as indicated by higher SF-36 physical summed and pain scores. From a different perspective, the subjectively perceived burden of headache of otherwise healthy individuals seems to exceed its assumed one, exerting a negative influence also on sexual life. So far, a substantial negative impact of headache has been shown on social and family life, careers and earnings of headache patients [35]. We cannot comment on the importance of headache attack frequency or severity because these data were not systematically collected; however, the results of Eraslan et al. [13] suggest that sexual dysfunction in headache patients is not related to attack frequency or severity.

Interpretation of our study data is limited because the sample size was small due to serious recruitment problems, most probably related to the subject of the study and study methodology requiring face to face contact with the patients in the setting of an outpatient clinic. Thus, the study may be biased towards women with a more open attitude to sexual life. In addition, we cannot exclude that women with sexual dysfunction were more likely to participate in this study. If such bias occurred, it most likely affected the two study groups similarly. Providing more information on sexual dysfunctions to both clinical physicians and patients could help to remove any taboos and, thus, to overcome recruitment problems in future studies. Another limitation concerns MSISQ-19, which was not validated for migraine patients, who were however instructed to think about their migraine when answering the questionnaire. Furthermore, MSISQ-19 made it unfeasible to examine a healthy control group. Finally, our findings cannot be transferred to the general population, as they were recorded in a tertiary centre.

Despite these limitations, the results of our study suggest that sexual dysfunction is not only a major problem in female patients with MS but also in women with migraine. The frequency of primary, secondary and tertiary sexual dysfunctions is similar both in MS and headache patients. Given the fluid nature of the three dimensions of sexual dysfunction as defined by Sanders [10], a clear allocation to one of these levels seems difficult. Comorbid depression was found in almost all patients, seems to be strongly associated with sexual dysfunction and may mimic primary sexual dysfunction. We therefore propose that diagnosis of sexual dysfunction should only be made after exclusion or successful treatment of comorbid depression.
